# The lncRNA HNF1A‐AS1 is a negative prognostic factor and promotes tumorigenesis in osteosarcoma

**DOI:** 10.1111/jcmm.12944

**Published:** 2017-09-02

**Authors:** Lijun Cai, Jinhan Lv, Yinquan Zhang, Junhong Li, Yinong Wang, Huilin Yang

**Affiliations:** ^1^ Department of Orthopedics the First Affiliated Hospital Soochow University Suzhou China; ^2^ Department of Orthopedics Ningxia People's Hospital Yingchuan Ningxia China; ^3^ Department of Orthopedics the Third Affiliated Hospital Second Military Medical University Shanghai China

**Keywords:** long noncoding RNA, HNF1A‐AS1, osteosarcoma, biomarker, function

## Abstract

Recent studies have revealed that long noncoding RNA HNF1A‐antisense 1 (HNF1A‐AS1) plays an important role in the development of several human malignancy entities. However, the expression and function of HNF1A‐AS1 in the carcinogenesis and development of osteosarcoma remains unknown. In this study, we detected the HNF1A‐AS1 levels in human osteosarcoma tissues and cell lines by quantitative real‐time polymerase chain reaction (qRT‐PCR), and investigated its role in osteosarcoma by using *in vitro* assays. Our study showed that HNF1A‐AS1 expression was significantly up‐regulated in human osteosarcoma tissues and cell lines compared with their normal counterparts, and its expression level was positively correlated with the distance metastasis (*P* = 0.009) and tumour stage (*P* = 0.019). Moreover, Kaplan–Meier curves with the log‐rank test showed that higher expression of HNF1A‐AS1 conferred a significantly poorer survival and multivariate Cox proportional hazards analysis revealed that HNF1A‐AS1 was an independent risk factor of overall survival. In addition, the expression of HNF1A‐AS1 in serum is correlated with patients’ status and receiver operating characteristic (ROC) curve analysis demonstrated that HNF1A‐AS1 could distinguish patients with osteosarcoma from healthy individuals (the area under curve 0.849, *P* < 0.001). Furthermore, *in vitro* knockdown of HNF1A‐AS1 by siRNA significantly inhibited cell proliferation and G_1_/S transition, and suppressed migration and invasion by reducing the epithelial‐mesenchymal transition (EMT) program in osteosarcoma cells. Taken together, our data suggested that HNF1A‐AS1 is a novel molecule involved in osteosarcoma progression, which may provide as a potential diagnostic, prognostic biomarker and therapeutic target.

## Introduction

Osteosarcoma is one of the most common type of primary sarcoma of the bone cancer in orthopaedics 1, 2. Although wide tumour excision is effective on primary disease, in addition to currently widely adopted adjuvant chemotherapy and radiotherapy on metastatic lesions, the cure rate of osteosarcoma patients remain dismal 3, 4. Therefore, understanding the exact molecular mechanisms underlying the histological heterogeneity, development of metastasis and drug resistance is essential for illustrating the molecular pathogenesis of osteosarcoma, as well as developing novel targets for the diagnosis, prognosis and treatment of osteosarcoma.

Long non‐coding RNA (lncRNA) belong to a class of RNA molecular that is longer than 200 nucleotides and cannot be translated into a protein, which take part in the control of gene and protein function through multiple mechanisms including the genetic, transcriptional and post‐transcriptional level 5. LncRNA is implicated in cell fate determination and human disease pathogenesis 6, 7. Particularly, the dysregulation of lncRNAs could play important roles in many types of cancer including osteosarcoma 8. For example, the expression level of lncRNA HOTAIR was over‐expressed in osteosarcoma tissues, and a SNP located in HOTAIR gene (rs7958904) was significantly associated with decreased risk of osteosarcoma 9. In addition, some studies have demonstrated that abnormal expression of various lncRNAs, such as H19 10, loc285194 11 and MALAT1 12, are functionally participated in osteosarcoma carcinogenesis and progression. However, the role of lncRNAs in osteosarcoma tumour development has only recently been investigated and remains largely unknown 13.

LncRNA HNF1A antisense RNA 1 (HNF1A‐AS1) is located on chromosome 12q24.31, a region frequently altered in renal cell carcinoma 14, cutaneous T‐cell lymphoma 15, neuroblastoma 16 and oesophageal carcinoma 17. Previous studies identified HNF1A‐AS1 as a functional lncRNA—HNF1A‐AS1 was significantly up‐regulated in oesophageal adenocarcinoma and lung adenocarcinoma tissues; increased HNF1A‐AS1 expression could regulate cell proliferation and metastasis in lung adenocarcinoma 18, while knockdown of HNF1A‐AS1 significantly inhibited cell proliferation, migration and invasion in oesophageal adenocarcinoma 19. Recently, by using microarray analysis, HNF1A‐AS1 was found up‐regulated in human osteosarcoma 13. However, little is known about the association between HNF1A‐AS1 expression levels and carcinogenesis and prognosis, in osteosarcoma.

In this study, we investigated the role of lncRNA HNF1A‐AS1 in human osteosarcoma. First, we investigated the expression of HNF1A‐AS1 in human osteosarcoma tissues, serum and cell lines by qRT‐PCR. Second, the correlation of HNF1A‐AS1 expression in osteosarcoma tissues with clinicopathologic features and prognosis in, as well as different disease status in serum, were analysed. Finally, *in vitro* cell biological assays in combination with small interfering RNA (siRNA)‐mediated knockdown were performed to probe the functional relevance of HNF1A‐AS1 in osteosarcoma.

## Materials and methods

### Patients and patient samples

Between 2007 and 2012, Osteosarcoma tissues and adjacent non‐tumour tissues were obtained from 72 osteosarcoma patients undergoing surgery at department of orthopaedic, the First Affiliated Hospital of Soochow University. None of the patients received preoperative chemotherapy or radiotherapy, or blood transfusion before surgery. All tissue samples were immediately snap frozen in liquid nitrogen after surgery and stored at −80°C until RNA extraction. The use of the tissue samples for all experiments was approved by Ethics Committee of The First Affiliated Hospital of Soochow University. Written informed consent was obtained from all participants. All patients’ slides were reviewed to confirm the diagnosis and to classify the tumour according to the sixth edition of the tumor node metastases (TNM) classification of the International Union Against Cancer (UICC). Patients with evidence of other malignancies or diseases were excluded from this study. Forty‐three patients completed the standard therapeutic regimen including surgical resection with wide or radical margin followed by adjuvant chemotherapy. The clinicopathologic characteristics of patients, including age, sex, alkaline phosphatase (ALP), tumour size, tumour location, initial metastasis, post‐operative chemotherapy and tumour stage are described in Table 1.

**Table 1:** The relationship between HNF1A‐AS1 expression and clinicopathologic parameters

Characteristics	No. of patients	Low expression (%)	High expression (%)	*P* value
Age (years)				
≤25	44	23 (52.3)	21 (47.7)	0.534
>25	28	13 (46.4)	15 (53.5)	
Gender				
Male	30	16 (53.3)	14 (46.7)	0.215
Female	42	20 (47.6)	22 (52.4)	
Tumour size (cm)				
≤8	34	18 (52.9)	16 (47.1)	0.143
>8	38	18 (47.4)	20 (52.6)	
Tumour location				
Tibia/Femur	49	25 (51.0)	24 (49.0)	0.311
Elsewhere	23	11 (47.8)	12 (52.2)	
Metastasis				
Yes	42	27 (64.3)	15 (35.7)	0.009^a^
No	30	9 (30.0)	21 (70.0)	
ALP				
Normal	39	18 (46.1)	21 (53.8)	0.128
Abnormal	33	18 (54.5)	15 (45.5)	
Chemotherapy				
Yes	43	25 (58.1)	18 (41.9)	0.031^a^
No	29	11 (37.9)	18 (62.1)	
Tumour stage				
IIA	32	19 (59.4)	13 (40.6)	0.019^a^
IIB/III	40	17 (42.5)	23 (57.5)	

ALP, alkaline phosphatase.

a *P* < 0.05.

We collected 30 blood samples in patients with osteosarcoma at 1 hr before surgery and 7 days after surgery, respectively. Meanwhile, 23 samples were obtained from patients after systemic post‐operative treatment, and 22 samples from patients who without any post‐operative treatment had experienced recurrence during follow‐up period. Twenty‐four patients with benign bone tumour (six cases of osteoclastoma, six cases of osteoidosteoma, six cases of osteochondroma and six cases of chondroblastoma) were collected. In addition, 21 age‐ and sex‐matched healthy individuals were enrolled as the control group, and they were underwent medical examination to exclude the evidence of tumour. All sera were collected using standard procedures.

### Cell culture, reagents and transfection

Human HOS, U2OS, SAOS‐2, MG63, SOSP‐9607 and 143B osteosarcoma cell lines, and the human osteoblast hFOB1.19 and NHOst were purchased from Shanghai Ruilu Technology Co. (Shanghai, China). Cells were cultured in Dulbecco's modified Eagle medium (DMEM)/high glucose supplemented with 10% foetal bovine serum (FBS; Gibco, Carlsbad, CA, USA) and 1% penicillin and streptomycin (Invitrogen, Carlsbad, CA, USA). And cells were incubated in a humidified incubator at 37°C with 5% CO_2_.

Anti‐GAPDH (sc‐365062), anti‐Vimentin (sc‐ 373717), anti‐N‐cadherin (sc‐393933), anti‐E‐cadherin (sc‐7870), mouse monoclonal antibodies and the horseradish peroxide‐conjugated mouse IgG secondary antibody (sc‐2025) were purchased from Santa Cruz Biotechnology, Inc. (Dallas, TX, USA). Two different anti‐human siRNAs of HNF1A‐AS1 and scramble siRNA, purchased from Life Technologies (Carlsbad, California, USA) were used: HNF1A‐AS1 siRNA1 (Cat. no. n265374), HNF1A‐AS1 siRNA2: (Cat. no. n265377). Cells were transfected with HNF1A‐AS1 siRNAs or scramble using Lipofectamine 3000 (Invitrogen) according to the manufacturer's protocol.

### Cell proliferation assay and cell cycle analysis

The cell proliferation assay was evaluated using a Cell Counting Kit‐8 assay (CCK‐8; Dojindo, Gaithersburg, MD, USA) according to the manufacturer's protocol. Briefly, 3 × 10^3^ cells/well incubated in 100 μl culture medium in 96‐well plates and treated with 10 μl/well of CCK‐8 solution during the last 4 hr of culture. The absorbance at 450 nm was measured in a ThermoMax Microplate Reader (Molecular Devices, Sunnyvale, CA, USA).

For cell cycle analysis, after transfection for 48 hrs, cells were harvested and fixed with ethanol overnight, then stained with propidium iodide (PI; Calbiochem, San Diego, CA, USA), and examined with a fluorescence‐activated cell sorting (FACS) flow cytometer (Beckman Coulter, Pasadena, CA, USA).

### Cell migration and invasion assay

Cells were grown to 70–80% confluence in 12‐well plastic dishes and were treated with si‐HNF1A‐AS1 or si‐NC. Then, 24 hrs after transfection, for migration assays, 1 × 10^5^ cells in serum free media were seeded in Transwell migration chambers (8 μm pore size; Millipore, Billerica, MA). For invasion assays, the upper chamber of these Transwell inserts was coated with Matrigel (Sigma, St. Louis, MO, USA ). Medium containing 20% FBS was added to the lower chamber. After 24 hrs, the non‐migrated or non‐invading cells were removed with cotton wool. Migrated or invasive cells located on the lower surface of the chamber were fixed with methanol, stained with Giemsa stain (Sigma) and then were counted using a microscope (Olympus, Tokyo, Japan) at 200× magnification. Each experiment was performed in triplicate.

### RNA extraction and qRT‐PCR analyses

Total RNA was isolated from tissues and cells by Trizol reagent (Invitrogen), cDNA was synthesized from 1 μg total RNA using a Reverse Transcription kit (Invitrogen Life Technologies), according to the manufacturers’ instructions. qPCR was performed with an ABI 7900 Real‐Time PCR System (Applied Biosystems Life Technologies, Foster City, CA, USA), using the following protocol: 95°C for 3 min., 40 cycles of 95°C for 15 sec., 60°C for 15 sec. and 72°C for 30 sec. The results were normalized to the expression of GAPDH. The primers used were as follows: HNF1A‐AS1 (forward primer, 5′‐TCAAGAAATGGTGGCTAT‐3′, and reverse primer, 5′‐GCTCTGAGACTGGCTGAA‐3′); MALAT1 (Forward primer, 5′‐GACGGAGGTTGAGATGAAGC‐3′, and reverse primer, 5′‐ATTCGGGGCTCTGTAGTCCT‐3′); GAPDH (forward primer, 5′‐CATCACCATCTTCCAGGAGCG‐3′, and reverse primer, 5′‐TGACCTTGCCCACAGCCTT‐3′).

### Western blot analysis

The cells lysates were added with sample buffer and boiled at 95°C for 5 min. Protein samples were separated using 10% sodium dodecyl sulphate‐polyacrylamide gel electrophoresis (SDS‐PAGE) and transferred onto nitrocellulose membranes. The membranes were blocked by 5% BSA for 30 min. and incubated with the primary antibodies at 4°C overnight, followed by incubation with secondary antibodies. Proteins were visualized using an enhanced chemiluminescence method kit (GE Healthcare Life Sciences, Piscataway, NJ, USA), and the protein band intensity was quantified *via* densitometric analysis using Quantity One software (Bio‐Rad Laboratories, Inc., Hercules, CA, USA).

### Statistical analysis

All statistical analysis was performed with SPSS, version 22.0 (SPSS, Inc., Chicago, IL, USA). Each experiment was repeated three times. The chi‐square and *t* tests were performed to explore the association between the HNF1A‐AS1 expression level and the clinicopathologic features. ROC curve was constructed for serum HNF1A‐AS1 and ALP levels, and the area under curve (AUC) was calculated to compare the ability of prediction for patients with osteosarcoma and healthy individuals. Overall survival curves was calculated by using Kaplan–Meier method, with the log‐rank test. The Cox proportional hazards model was used in the univariate and multivariate analysis. All tests were two‐sided, and a *P* value <0.05 was considered to be statistically significant.

## Results

### HNF1A‐AS1 is up‐regulated in osteosarcoma tissues and correlates with poor prognosis

**Figure 1:**
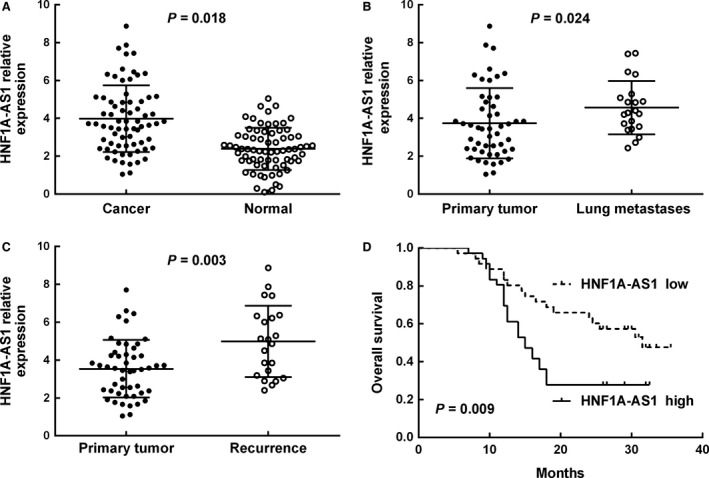
HNF1A‐AS1 was highly expressed and predicted poor prognosis in osteosarcoma. (**A**) Relative expression of HNF1A‐AS1 in osteosarcoma tissues in comparison with adjacent non‐tumour tissues. (**B**) Relative expression of HNF1A‐AS1 in tissues of osteosarcoma patients with primary lesion in comparison with patients with lung metastases. (**C**) Relative expression of HNF1A‐AS1 in tissues of osteosarcoma patients with primary lesion in comparison with patients with recurrence. (**D**) Kaplan–Meier overall survival curves of patients with osteosarcoma based on HNF1A‐AS1 expression (low *vs*. high, log‐rank test).

To evaluate the expression of lncRNA HNF1A‐AS1 in osteosarcoma, we conducted qRT–PCR in 96 patients with osteosarcoma. Our results showed that HNF1A‐AS1 expression in human osteosarcoma tissues was significantly higher than that in adjacent non‐tumour tissues (Fig. 1A, *P* = 0.018). In addition, the expression level of HNF1A‐AS1 was significantly higher in lung metastases compared with paired primary osteosarcoma (*P* = 0.024, Fig. 1B). Furthermore, increased expression of HNF1A‐AS1 was observed in local recurrences compared with paired primary osteosarcoma (*P* = 0.003, Fig. 1C).

Based on the expression levels of HNF1A‐AS1 obtained by qRT–PCR, we divided the 72 osteosarcoma patients into a high‐HNF1A‐AS1 expression group and a low‐HNF1A‐AS1 expression group according to the median expression level of HNF1A‐AS1 20. The associations between clinicopathologic characteristics and HNF1A‐AS1 expression were summarized in Table 1. Our results showed that high HNF1A‐AS1 expression level was correlated with tumour metastasis (*P* = 0.009), chemotherapy (*P* = 0.031) and tumour stage (*P* = 0.019). However, high HNF1A‐AS1 expression was not associated with other clinicopathologic characteristics of osteosarcoma patients, including age, gender, tumour size, tumour location, as well as serum ALP level (*P* > 0.05, Table 1).

When linked to the prognosis, the Kaplan–Meier method and log‐rank test showed that patients with high levels of HNF1A‐AS1 expression in osteosarcoma tissues had significantly shorter overall survival (*P* = 0.009, Fig. 1D) than those with low levels. Univariate Cox proportional hazards regressions model analysis demonstrated that distant metastasis, serum ALP level, concurrent chemotherapy, tumour stage and HNF1A‐AS1 expression were correlated with the overall survival of osteosarcoma patients (Table 2). Multivariate analysis using the Cox proportional hazard model confirmed that distant metastasis, tumour stage and HNF1A‐AS1 expression were independent prognostic factors for overall survival (Table 2). These findings indicated that overexpression of HNF1A‐AS1 may contribute to the pathogenesis and progression of osteosarcoma.

**Table 2:** Univariate and multivariate analysis of different prognostic factors for overall survival in 72 patients with osteosarcoma

Prognostic factors	HR	95% CI	*P* value	HR	95% CI	*P* value
Age (≤25/>25)	0.960	0.527–1.748	0.893			
Gender (male/female)	1.011	0.527–1.938	0.975			
Tumour size (≤8 cm/>8 cm)	0.871	0.271–2.801	0.816			
Tumour location (tibia, femur/elsewhere)	0.823	0.394–1.716	0.603			
Pathological type (telangiectatic, small cell/others)	1.138	0.605–2.143	0.688			
Metastasis (yes/no)	2.262	1.238–4.133	0.008^a^	2.643	1.400–4.956	0.003^a^
ALP (Normal/Abnormal)	2.785	1.398–5.545	0.004^a^			
Chemotherapy (Yes/No)	2.927	1..436–5.964	0.003^a^			
Tumour stage (IIA /IIB, III)	6.204	2.206–17.449	0.004^a^	6.521	2.299–18.491	0.000^a^
HNF1A‐AS1 (high /low)	2.258	1.220–4.179	0.009^a^	2.634	1.550–5.647	0.001^a^

HR, hazard ratio; CI, confidence interval; ALP, alkaline phosphatase.

a *P* < 0.05.

### Serum HNF1A‐AS1 levels correlate with disease status of osteosarcoma patients

**Figure 2:**
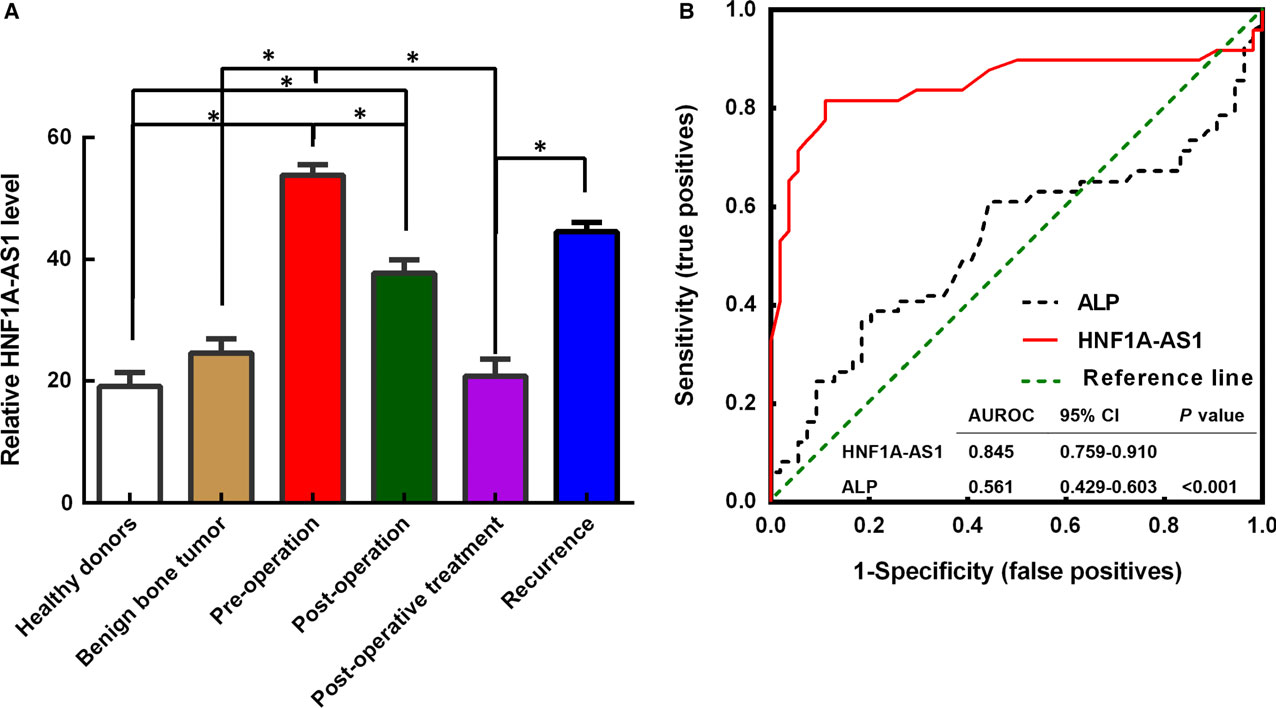
Serum HNF1A‐AS1 levels can indicate disease status of osteosarcoma patients. (**A**) The serum HNF1A‐AS1 levels in healthy donors, patients with benign bone tumour, pre‐operative and post‐operative osteosarcoma patients, osteosarcoma patients with post‐operative chemotherapy, and recurrence. *P < 0.05. (**B**) ROC curves of the plasma HNF1A‐AS1 and ALP in 30 newly diagnosed patients and 21 healthy donors.

To investigate whether the expression level of HNF1A‐AS1 change along with disease status of osteosarcoma patients, HNF1A‐AS1 relative expression levels were detected in the sera of pre‐operative and post‐operative osteosarcoma patients, and healthy donors. Our results suggested that HNF1A‐AS1 expression levels were obviously higher in the pre‐operative patients (53.83 ± 1.76) compared with the healthy donors (20.78 ± 2.79; *P* < 0.01), or the post‐operative patients (37.71 ± 2.23; *P* < 0.01) (Fig. 2). This suggested that HNF1A‐AS1 overexpression was derived from osteosarcoma. The HNF1A‐AS1 expression levels was further explored in patients with benign bone tumour, osteosarcoma patients underwent post‐operative chemotherapy, or with tumour recurrence. Our results indicated that the expression levels of HNF1A‐AS1 were significantly decreased in patients with post‐operative chemotherapy (19.14 ± 2.21) to a level that was similar to that of healthy donors and patients with benign bone tumour (23.63 ± 2.27). Furthermore, HNF1A‐AS1 expression was obviously elevated in patients with disease relapse (44.52 ± 1.55) compared with the post‐operative treated patients (*P* < 0.01). Our results showed that HNF1A‐AS1 expression was changed dynamically and associated with disease status in osteosarcoma.

### Diagnostic value of HNF1A‐AS1 in patients with osteosarcoma

To further evaluate whether HNF1A‐AS1 in sera can be a potential diagnostic biomarker for patients with osteosarcoma, a ROC curve using serum ALP and HNF1A‐AS1 was established for 30 osteosarcoma patients (by using the serum collected at 1 hr before surgery) and 21 healthy individuals. As shown in Fig. 2B, the AUC for HNF1A‐AS1 was 0.845 [95% confidence interval (CI), 0.759–0.910], with a sensitivity of 87.2% and a specificity of 88.9%, and in our cohort, the AUC for ALP was 0.561, with a sensitivity of 34.6% and a specificity of 88.2%, the diagnostic power of HNF1A‐AS1 was more effective than ALP at distinguishing osteosarcoma from healthy individuals (*P* < 0.001).

### Suppressing HNF1A‐AS1 expression decreases osteosarcoma cell growth and metastasis

**Figure 3:**
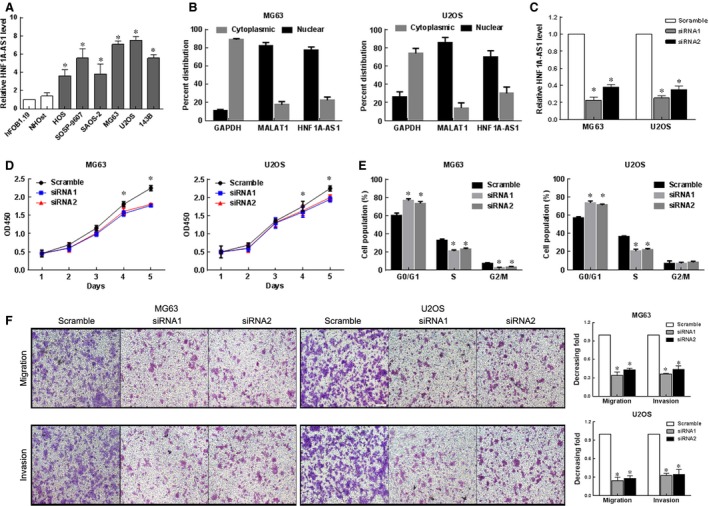
Down‐regulated HNF1A‐AS1 inhibited cell migration, and invasion of osteosarcoma cells. (**A**) qRT‐PCR results of HNF1A‐AS1 mRNA level in six osteosarcoma cell lines and one normal human bone cell line. *P < 0.05. (**B**) HNF1A‐AS1 nuclear localization, as identified using qRT‐PCR in fractionated MG63 and U2OS cells. GAPDH was used as a cytoplasmic marker and MALAT1 was used as a nucleus marker. (**C**) The HNF1A‐AS1 levels in MG63 and U2OS cells transfected with HNF1A‐AS1‐siRNAs or Scramble detected by qRT‐PCR. *P < 0.01. (**D**) CCK‐8 assays revealed cell growth curves of indicated cells. *P < 0.05. (**E**) Flow‐cytometric determination of proportion of indicated cells in distinct cell‐cycle phases. *P < 0.05. (**F**) Representative images (left) and quantification (right) of Transwell migration and invasion assays for MG63 and U2OS cells (scale bars = 50 μm). *P < 0.01.

To investigate the baseline expression level of HNF1A‐AS1, we conducted qRT–PCR in six osteosarcoma cell lines (HOS, U2OS, SAOS‐2, MG63, SOSP‐9607 and 143B) and two human osteoblasts (hFOB1.19 and NHOst). And HNF1A‐AS1 was up‐regulated in all of the six analysed osteosarcoma cell lines compared with the human osteoblasts (all *P* < 0.05, Fig. 3A). The MG63 and U2OS cell lines exhibited the highest HNF1A‐AS1 expression levels and were thus chosen for the subsequent *in vitro* knock‐down experiments. We next measured the nuclear and cytoplasmic expression level of HNF1A‐AS1 in MG63 and U2OS cells by qRT‐PCR. The differential enrichments of GAPDH and MALAT1 were used as fractionation indicators. We observed a considerable higher in HNF1A‐AS1 expression in the nucleus *versus* the cytoplasm (Fig. 3B).

To explore the role of HNF1A‐AS1 in development of osteosarcoma, MG63 and U2OS cells were transfected with HNF1A‐AS1‐siRNAs or Scramble with high transfection efficiency (Fig. 3C, *P* < 0.05). Then, the effects of HNF1A‐AS1 on the cell growth of osteosarcoma cells were checked by CCK‐8 assay. As shown in Fig. 3D, the proliferation of MG63 and U2OS cells was significantly reduced after knockdown of HNF1A‐AS1 (*P* < 0.05). Accompanying with this inhibition of cell proliferation, our result showed a significant increase in the percentages of cells in G1‐phase but decreased proportions of S‐phase cells in both HNF1A‐AS1‐siRNAs transfected MG63 and U2OS cells (Fig. 3E).

To investigate whether declined expression of HNF1A‐AS1 can affect the migration and invasion of osteosarcoma cells, we examined the rate of HNF1A‐AS1‐siRNAs transfected MG63 and U2OS cells migration by Transwell assays. As shown in Fig. 3F, the migration and invasion capacity of MG63 and U2OS cells was significantly reduced after knockdown of HNF1A‐AS1 (*P* < 0.05).

### HNF1A‐AS1 silencing influence the expression of EMT‐related proteins in osteosarcoma cells

**Figure 4:**
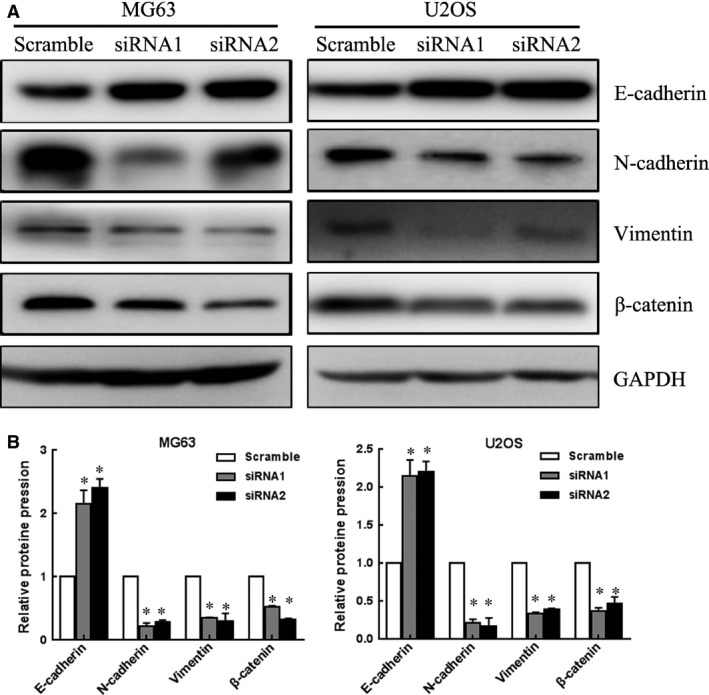
HNF1A‐AS1 silencing influence the expression of EMT‐related proteins in osteosarcoma cells. (**A**) Analysis of E‐cadherin, N‐cadherin, vimentin and β‐catenin expression in MG63 and U2OS cells treated with HNF1A‐AS1 siRNA by Western blot. GAPDH was used as an internal control. (**B**) Results shown are the mean ± SD of repeated independent experiments. Quantification was normalized to GAPDH. *P < 0.05.

To investigate the potential mechanisms underlying the poor migration and invasion of HNF1A‐AS1 knockdown cells, we analysed the relative levels of EMT‐related molecules and metastatic regulators in HNF1A‐AS1 siRNAs and Scramble transfected cells by western blot assay. The result showed that the relative levels of N‐cadherin, Vimentin and β‐catenin expression were significantly lower in HNF1A‐AS1 siRNAs transfected cells than that in Scramble transfected cells, whereas the levels of E‐cadherin were significantly higher (Fig. 4, *P* < 0.05). Together, these results indicate that HNF1A‐AS1 knockdown inhibits the malignant phenotypes of osteosarcoma cells, which might be attributed to the regulation of EMT‐related proteins expression.

## Discussion

In this study, we explored the expression of lncRNA HNF1A‐AS1 and its biological functions in osteosarcoma. We confirmed that HNF1A‐AS1 was overexpressed in osteosarcoma. Clinically, high expression levels of HNF1A‐AS1 correlated with worse survival of osteosarcoma patients, indicating that HNF1A‐AS1 might be a novel prognostic indicator for patients with osteosarcoma. In addition, the expression of HNF1A‐AS1 in sera is correlated with disease status and may serve as a molecular indicator for osteosarcoma diagnosis, as well as evaluating therapeutic efficacy. Furthermore, our *in vitro* experiments indicated that HNF1A‐AS1 contributes to osteosarcoma cell growth by promotes G_1_/S transition, and induces metastasis of osteosarcoma via induction of EMT.

The lncRNA HNF1A‐AS1 (NR_024345) was firstly found as a strongly over‐expressed non‐coding transcripts (2455 bases in humans) in oesophageal adenocarcinoma, and implicated in various of malignant biological behaviors of oesophageal adenocarcinoma cells 19. Recently, by microarray analysis, Li and colleagues identified that HNF1A‐AS1 was also up‐regulated in osteosarcoma 13. However, the possible role and associated molecular mechanisms of HNF1A‐AS1 in osteosarcoma are yet to be clarified. By qRT‐PCR, this study verified that the expression level of HNF1A‐AS1 were up‐regulated in osteosarcoma tissues and cells compared with their normal counterparts, suggesting that the overexpression of HNF1A‐AS1 may also be an important molecular event in osteosarcoma.

The correlation of HNF1A‐AS1 overexpression with a higher tumour burden including presence of distance metastasis and advanced TNM stage in our study, suggesting that HNF1A‐AS1 may accelerate tumour progression in osteosarcoma mainly *via* stimulating malignant phenotype of osteosarcoma cells, which was supported by the results of knockdown of HNF1A‐AS1 in MG63 and U2OS cells. Considering that the clinical stage was majorly concerned about tumour growth and metastasis, it was not surprising that the expression of HNF1A‐AS1 was tightly correlated with tumour stage. Indeed, HNF1A‐AS1 might push the advance of clinical stage by inducing tumour growth, migration and invasion in osteosarcoma. Therefore, it was reasonable that overexpression of HNF1A‐AS1 confer poorer survival in osteosarcoma patients, and it was considered to be an independent prognostic indicator for overall survival. Taken together, monitoring the levels of HNF1A‐AS1 might be an effective biomarker for the prognosis prediction of osteosarcoma.

It is well known that lncRNAs are detectable in circulating system of cancer patients, given the versatile roles played by lncRNAs in human malignancies, it is not surprising that the expression levels of lncRNAs may be indicators of the intrinsic characteristics of tumours, and therefore may be utilized as a tool for cancer diagnosis and disease status monitoring 21, 22. In this study, we collected sera of osteosarcoma patients in different disease status, patients with benign bone tumour and healthy donors, then compared the expression levels of HNF1A‐AS1 among these subgroups. We found that the serum HNF1A‐AS1 levels were higher in pre‐operative patients compared with healthy donors and patients with benign bone tumour, and similar results were observed in post‐operative patients. Furthermore, its expression levels were elevated again in patients who had experienced disease recurrence. These results indicated that serum HNF1A‐AS1 levels was closely associated with disease status and could be used to monitoring disease status. In addition, we also assessed the ability of serum HNF1A‐AS1 in distinguishing osteosarcoma from healthy individuals. Our results demonstrated that the diagnostic performance of HNF1A‐AS1 was significantly higher than that of ALP, indicate that serum HNF1A‐AS1 should be further evaluated as novel non‐invasive biomarkers for discriminating osteosarcoma from benign bone tumours or healthy individuals. As a widely useful diagnostic biomarker for osteosarcoma patients, ALP is found to confer good specificity and sensitivity for the diagnosis of osteosarcoma, whereas its use in teenagers is problematic because ALP levels are affected by age, gender and pubertal stage 23. Whether this short board is also meet by serum HNF1A‐AS1, or HNF1A‐AS1 is an independent molecular indicator, need for further large‐scale and multi‐centre studies to verify.

Furthermore, we studied the effect of lncRNA HNF1A‐AS1 on the biological function of osteosarcoma cells, and found that knockdown of HNF1A‐AS1 significantly reduced osteosarcoma proliferation, suppressed S‐phase entry and inhibited migration and invasion *in vitro*. Mechanically, HNF1A‐AS1 function as an oncogene in osteosarcoma through regulation of EMT‐related proteins. Previous studies have shown that several cancer‐related gene, such as H19 and DNMT1, exhibits correlation with HNF1A‐AS1, which may play roles in the HNF1A‐AS1‐mediated inhibition of cancer progression 18, 19. It has been proved that HNF1A‐AS1 may mediate the binding of DNMT1 to E‐cadherin which decreases the E‐cadherin and induces EMT in lung adenocarcinoma 18. However, as the molecular mechanism of one lncRNA may differ between different tissues and cell types, HNF1A‐AS1 might regulate osteosarcoma cell tumorigenesis and metastasis through different potential targets, and the specific target genes controlled by HNF1A‐AS1 in osteosarcoma require further studies to illustrate.

## Conclusions

Our results suggested that lncRNA HNF1A‐AS1 expression was up‐regulated in osteosarcoma and was associated with the biological aggressiveness and progression of osteosarcoma. Moreover, the expression of HNF1A‐AS1 in sera is correlated with patients’ status and may serve as a novel biomarker in osteosarcoma diagnosis, as well as disease status monitoring. Furthermore, knockdown of HNF1A‐AS1 could inhibit cell proliferation and suppress G1/S transition, reduce the migration and invasion of osteosarcoma cells *in vitro* by induce EMT program. These findings demonstrated that HNF1A‐AS1 could serve as a potential biomarker and therapeutic target for osteosarcoma.

## Conflict of interest

The authors declare no conflict of interest.
